# Tuberculosis of the breast: analysis of 17 cases

**DOI:** 10.11604/pamj.2020.37.282.26583

**Published:** 2020-11-26

**Authors:** Amal Bouziyane, Hicham Benaguida, Maryame Lamsisi, Ayoub Khoaja, Samira Benayad, Mohammed Ennachit, Mohamed Elkarroumi, Mustapha Benhessou, Moulay Mustapha Ennaji

**Affiliations:** 1 Mohamed VI University of Health Sciences, Casablanca, Morocco,; 2 Laboratory of Virology, Microbiology, Quality and Biotechnologies/ETB, Faculty of Science and Techniques, Hassan II University of Casablanca, Mohammedia, Morocco,; 3 Mohammed VI Center for Cancer Treatment, University Hospital Ibn Rochd, Casablanca, Morocco,; 4 Department of Anatomopathology, University Hospital Ibn Rochd, Casablanca, Morocco

**Keywords:** Tuberculosis, mastopathy, breast cancer

## Abstract

Tuberculosis constitutes a major public health problem in the world. Certain extra-pulmonary locations of tuberculosis disease are very exceptional. Amongst these, tuberculosis of the breast is rare even in countries where this infection is endemic. This form of tuberculosis is characterized by clinical and radiological polymorphisms and might mimic other diseases, especially breast cancer. This retrospective study is entailing seventeen patients treated in the Onco-Gynecology Department of the Mohammed VI Cancer Treatment Center, in the Ibn Rochd University Hospital of Casablanca, for breast tuberculosis, over a period of three years. We report the epidemiological, clinical and paraclinical aspects and we specify the treatment and evolution of the patients.

## Introduction

Tuberculosis is a very old transmissible disease. However, its etiology remained unknown until March 24^th^, 1882, when Dr. Robert Koch announced his discovery of the bacillus subsequently named *Mycobacterium tuberculosis* [1,2]. Tuberculosis constitutes a major public health problem in the world. Globally, World Health Organization estimates that around 10.0 million (range 9.0-11.1 million) people became ill with tuberculosis in 2018, which remained relatively stable in recent years. The number of incident tuberculosis cases in Morocco reached 31,712 in 2018. In the same year, mortality linked to this disease was estimated at 3,000 deaths with a mortality rate of 8.2 per 100,000 inhabitants [3].

Breast is a rare localization of extra-pulmonary tuberculosis, particularly as a primary manifestation, even in endemic countries of tuberculosis [4]. Mammary tuberculosis was first described by Sir Astley Cooper in 1829 and since then, numerous studies on important series appeared and are all unanimous as to its rarity [5]. The incidence is estimated at 0.1% of breast disease in developed countries, but it reaches 3% to 4% in countries where tuberculosis is endemic such as India and Africa [6].

Tuberculosis is getting a renewed interest of researchers and clinicians because of the recent increase in its incidence in developed countries, in particular, extra-pulmonary tuberculosis. Considering the progressive character conferred to it by hormonal incentives, different from those of other locations, mammary tuberculosis mainly affects lactating multiparous women and in association with immuno-suppressive disorders, including Human Immunodeficiency Virus (HIV) infection. It is often confused both clinically and mammographically with a cancerous lesion which remains the first reflex of the clinician, but it can mimic a pyogenic abscess and other granulomatous diseases as well [7]. Our study aims to specify the epidemiological, clinical, paraclinical, therapeutic and progressive characteristics of breast tuberculosis and to take stock of the difficulties of the diagnosis and the management.

## Methods

In this retrospective study we enrolled seventeen patients treated for breast tuberculosis, over three years, starting from January 2017 to December 2019, in Department of Gynecology, University Hospital Cheikh Khalifa and the Onco-gynecology Department of Mohammed VI Center for Cancer Treatment, Ibn Rochd University Hospital of Casablanca, Morocco. For each case, the following parameters were collected from hospitalization and the follow-up data: epidemiological data: frequency, age, sex, genital activity status, parity, breastfeeding, history; clinical data: time before the consultation, the reason for the hospitalization, general signs, functional signs and physical signs; paraclinical data: biological assessment, radiological assessment, breast fine-needle aspiration, breast biopsy with the bacteriological and anatomopathological study; treatment: medical treatment; surgical treatment; evolution: relapse or recurrence, complete remission, women lost to follow-up, adverse effects observed during or after treatment.

## Results

**Epidemiological description of patients:** the frequency of breast tuberculosis was 0.64% of all mastopathies, the mean age of patients was 33.5 years, with extremes of 18 years and 54 years. All the patients in our series were female. Eleven patients were in a genital activity period, which constitute 64.70% of cases, 11.77% were in peri-menopause and 23.53% were in menopause. The majority of patients were multiparous, i.e. 59% of cases, 23% were nulliparous and 18% were primiparous. A total of 52.9% of cases had history of breastfeeding during an average period of 6 months. Overall, 70.6% of the patients had no particular medical or surgical pathological history, tuberculosis contagion was noted in 11.8% of cases, 2 patients treated for extra-pulmonary tuberculosis, i.e. 11.8% and 1 patient treated from breast abscess of a bacterial origin. The diagnosis was made in 41.2% of cases after six months from the onset of the symptoms, the deadline for consultation. Also, 58.85% of women consulted for a breast lump, 23.5% for breast lump with skin fistula and breast abscess in 17.65% of the cases. The demographic and clinical data of the patients are summarized in Table 1.

**Table 1:** demographic and clinical data of enrolled patients with mammary tuberculosis (N=17)

Variables		Frequency (%)
Genital activity	Genital activity period	64.70
	Peri-menopause	11.77
	Menopause	23.53
Parity	Multiparous	59
	Nulliparous	23
	Primiparous	18
Breastfeeding history	Yes	52.9
	No	47.1
Medical or surgical pathological history	Yes	29.4
	No	70.6
Tuberculosis contagion	Yes	11.8
	No	88.2
Treatment for extrapulmonary tuberculosis	Yes	11.76
	No	88.24
Treatment for from breast abscess	Yes	5.88
	No	94.12
Consultation reason	Breast lump	58.85
	Breast lump with skin fistula	23.5
	Breast abscess	17.65

**Figure 1:**
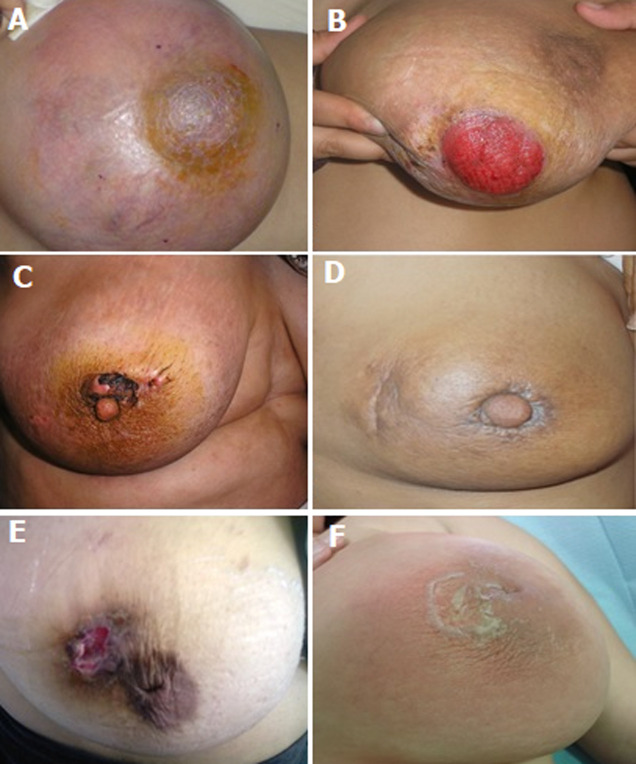
different clinical and physical presentation of patients with mammary tuberculosis: A) right breast abscess; B) an ulcer that involve and destroy the nipple-areola complex; C) an ulcer on the upper quadrant of the nipple-areola complex; D) scar of an ulcer on the upper outer quadrant of the right breast with a retracted nipple; E) an inverted nipple with an ulcer on the upper outer quadrant of her right breast; F) periareolar eczematiform lesion

**Clinical and paraclinical examination:** on clinical examination, 35.3% of patients had an altered general condition. Functional signs were absent in 58.83% of patients. A total of 29.41% of cases showed mastodynia and 11.76% of cases presented with breast discharge. On physical examination, a breast arch with redness was present in 29.4% of patients. The mean size of the swelling was 5.4 cm. There were 52.94% of the lesions located in the upper outer quadrant (Figure 1). Axillary lymphadenopathy was present in two patients in our series (76% of cases). One patient presented breast tuberculosis associated with pleuropulmonary and cerebral localization.

**Figure 2:**
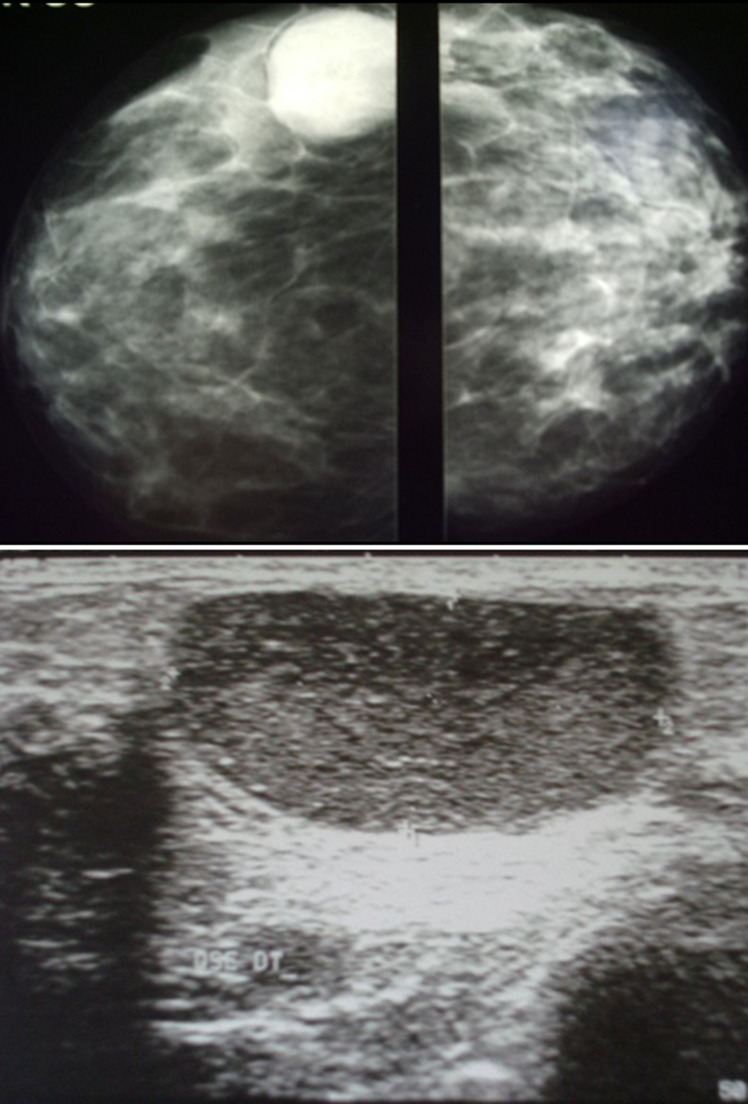
mammogram of the right breast showing regular opacity of the upper inner quadrant

**Figure 3:**
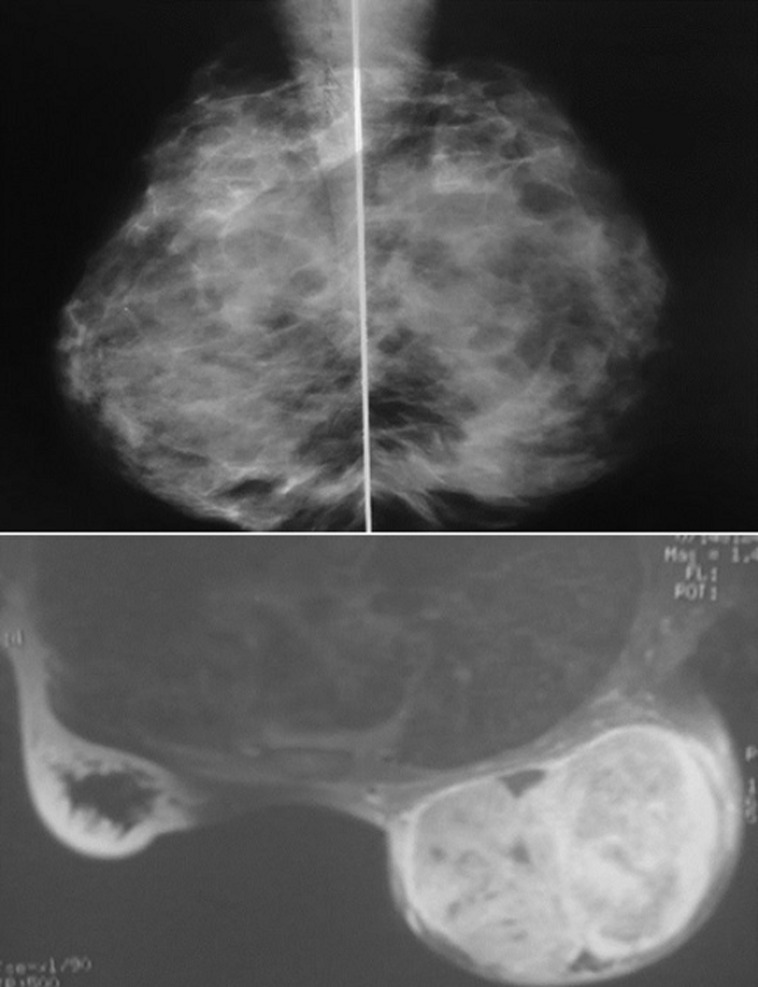
mammogram of the left breast, with transparent, fairly dense X-ray appearance; MRI appearance of a voluminous well-limited echogenic formation, with posterior reinforcement of the right breast of 127 x 110 x 103 mm

**Figure 4:**
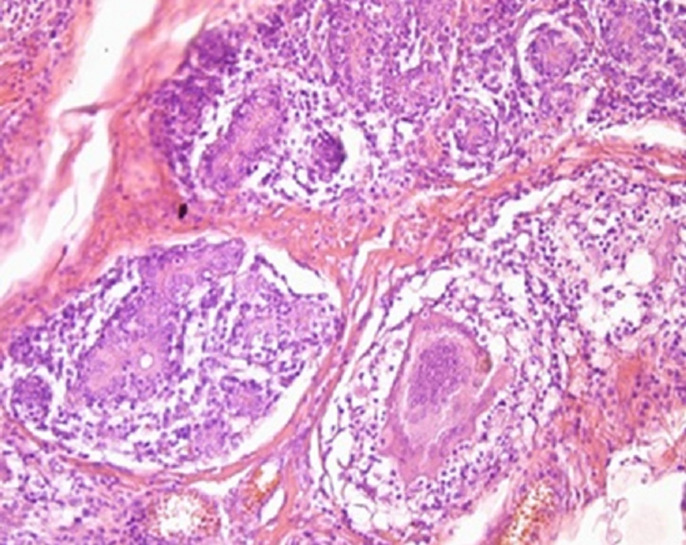
histological examination showing mammary parenchyma with cellular epithelia-giganto granuloma with caseous necrosis

Laboratory tests had shown an increase in Sedimentation Rate (ESR) in eight patients, positive intra dermic reaction in six patients, Koch bacillus test in sputum was positive in one patient and all patients had a normal blood count (CBC). On the radiological assessment, mammography was performed in 88.24% of cases. It had shown dense opacity with regular limits in 40% of cases. Increased opacity in 33.3% of cases. Dense breast in 13.3% of cases (Figure 2). Opacity with irregular limits in 6.7% of cases and skin thickening of the areolar plaque in 6.7% of cases. Breast ultrasound was performed in all patients, 81.25% of patients had an echogenic, anechoic or heterogeneous ultrasound image, well limited or poorly limited; the ultrasound image of associated axillary lymphadenopathy was shown in 18.75% of patients. Magnetic resonance imaging (MRI) was performed on a single patient in our series, showing the appearance of a large lesion of the right breast (Figure 3). The chest X-ray was normal in all patients. The breast biopsy confirming the diagnosis was made in all patients, the anatomo-pathological study found a histological appearance of a cellular epithelia-giganto granuloma with caseous necrosis (Figure 4); the bacteriological study of a product from the isolation of Koch bacillus was positive in 52.95% of cases.

**Treatment and patients' evolution:** the therapeutic management was based on an anti-bacillary treatment, including Isoniazid (H), Rifampicin (R) and Pyrazinamide (Z), 76.47% of patients received nine months of treatment, 23.53% of patients received 12-month treatment, surgical treatment resulted in abscess drainage in 35.29% of cases, lumpectomy in 17.65%, abscess drainage with resection of cubicles and lumpectomy in 17, 65% of cases.

The evolution has been generally good. Complete recovery at the end of treatment was observed in 64.70% of patients, recurrence was noticed in 23.53% of patients, 47.06% of patients did not represent complications during the treatment, three patients were lost to follow-up.

## Discussion

The mammary gland appears to have a particular resistance to tuberculosis infection like the skeletal muscles and the spleen, which makes it difficult for the survival and multiplication of tuberculosis bacilli [8]. The different modes of breast infection are the hematogenous route, the lymphatic route, spreading from contiguous structures such as infected ribs, costochondral cartilage, breastbone, shoulder joint or direct inoculation by skin abrasion nipples or through the milk ducts and ductal infection [9,10]. It usually affects young women between the ages of 20 and 40, multiparous, breastfeeding. It can also be reported in prepubescent men or older women.

In our series the mean age of patients was 33.5 years, the majority were multiparous and 9 patients breastfed. This may be due to physiological changes in the breast during the period of breastfeeding when the breast is susceptible to infections and trauma, namely due to increased blood supply, dilation of ducts during lactation and physiological stress of breastfeeding [11]. Some authors have considered that almost all cases of mammary tuberculosis were secondary, even if the primary location of the infection remains hidden. Rare cases of primary mammary tuberculosis are thought to be caused by infection of skin abrasions through the breast or the main ducts of the nipple [8]. The clinical presentation of breast tuberculosis is generally poorly described in the literature and clinically important features are not consistently reported. In most cases, the constitutional symptoms of tuberculosis (fever, weight loss, night sweats or deterioration in general condition) are rarely seen [12]. In our study, most patients were in good general condition, accounting for 64.7% of cases.

The onset progresses insidiously over an average duration varying from a few weeks in Europe [13,14] to several months, in India and sub-Saharan Africa [15]. The disease is usually unilateral and can also affect the other's breast [16]. There is a clear predilection of breast tuberculosis lesions for the outer quadrants, probably due to the proximity to the axillary region from which retrograde lymphatic spread [17,18].

The appearance of a lump is the most common presentation, with other less common forms such as cold abscess and diffuse inflammation of the breast. Diagnosis remains difficult, as it can simulate a large number of much more common conditions, especially in older women where breast cancer remains the primary concern. We also note breast fistulization or that of axillary lymphadenopathy associated with a breast tumor whose development is slow is strongly suggestive. By comparison, in the carcinoma of literature, studies have shown a gross nipple-areola involvement rate of 12.5% (41/326 cases) in Laronga *et al*. and 8% (99/1291 consecutive cases) in Santini *et al*. [19,20].

Breast tuberculosis can be classified into three types, namely: nodular, disseminated and sclerosing varieties. McKeown and Wilkinson classified breast tuberculosis into five different types [9]: A) nodular tuberculous mastitis; B) disseminated or concomitant tuberculous mastitis; C) sclerosing tuberculous mastitis; D) tuberculous obliterating mastitis; and E) acute miliary tuberculosis. The radiological assessment is based on the mammography which is an essential element in the radiological exploration even if it is not very specific where the images rather pointing towards a malignant etiology, sclerosing tuberculous mastitis reveals a dense homogeneous mass with fibrous septa and retraction of the nipple [21].

On ultrasound, a hypoechoic mass is found in 60% of patients and the method can sometimes identify a visible fistula or sinus in tuberculous mastitis [22]. It is used to assess whether the chest wall is affected by the lesion. Mammographic and ultrasound features of tuberculous mastitis in a study by Sakr *et al*. revealed mass lesions mimicking malignant tumors (30%), smooth lined masses (40%), axillary or intramammary lymphadenopathy (40%), asymmetric density and duct ectasia (30%), skin thickening and retraction of the nipple, macrocalcification (20% each) and cutaneous sinus (10%). On ultrasound, 60% of had hypoechoic masses, 40% of ectasias of the focal or sectoral canals and 50% of axillary lymphadenopathy [23]. The gold standard for the diagnosis of mammary tuberculosis is the detection of M. tuberculosis by Ziehl Neelsen stain or by culture. However, histo-chemistry is impractical and culture has limitations due to the delay in obtaining the result and the possibility of false negatives in paucibacillary. Fine needle aspiration cytology may not be able to detect the causative pathogen itself.

Anatomopathological analysis of a breast biopsy revealed epithelial-giant cellular granuloma with caseous necrosis in 73% to 95% of cases [17,24], a similar histological appearance can be found in other granulomatous affections of the breast; hence the need of standard bacteriological culture and mycobacteriology of part of the biopsy samples. The polymerase chain reaction (PCR) is very sensitive to the diagnosis of breast tuberculosis. Although rarely used, it is recommended in case of negative culture results or for the differential diagnosis between other forms of granulomatous mastitis. On the other hand, in various series, in most cases, the diagnosis was made based on pathology and confirmed in response to anti-tuberculosis treatment [25-28].

Current treatment is mainly medical using anti bacillary, since their discovery in 1944, The regimes using a triple therapy (HR, EB or HRZ) or a quadruple therapy (HR, EB, Z) for two to three months, followed by a double treatment (HR) for nine months in the case of initial triple therapy and six months in the case of initial quadruple therapy are sufficient to sterilize breast tuberculosis. In our series, the anti-bacillary regimen followed in all patients was triple therapy (HRZ) for two to three months, followed by dual treatment (HR) for a period of seven to nine months. The most common approach is the standard treatment of 2 months of Isoniazid, Rifampin, Pyrazinamide and Ethambutol, followed by 4 months of Isoniazid and Rifampicin. Some authors prefer the 9-month regimen (2 months of Isoniazid, Rifampicin, Pyrazinamide and Ethambutol and 7 months of Isoniazid and Rifampicin) because of an overall lower relapse rate [29].

Infection with multidrug-resistant tuberculosis has been reported and the continuation phase can be prolonged, usually to 12 months, but up to 18 months in slow clinical response cases [30]. In general, complete resolution is achieved in most patients. Surgical treatment has a dual purpose: to confirm the diagnosis of tuberculosis disease and to supplement medical treatment when necessary. There is a wide range of surgical techniques ranging from simple small surgical means including puncture, flattening, drainage or curettage of fistulas, to excisional surgery, whether it be a “simple lumpectomy” or a mastectomy. The effectiveness of a treatment combining chemotherapy and surgery in reducing the rate of recurrence compared to anti bacillary treatment alone deserves to be proven by a large statistical study [4].

The course of breast tuberculosis under well-managed early treatment is generally favorable in 89.7% of cases [31]. Left untreated, breast tuberculosis has an unfortunate prognosis, eventually invading the whole breast step by step, as well as the chest wall and pleural space. Recurrence may be local or at the level of the axillary hollow, even on the opposite side as a result of inadequate anti bacillary treatment. In our series, the evolution was good with a complete cure rate observed in the majority of patients, i.e. 64.70% of cases.

## Conclusion

Breast tuberculosis is a rare condition and its frequency remains low and is seen mainly during the period of genital activity. The lymphatic route is the most frequent route of contamination. The clinical and radiological pictures are often misleading and constitute a real problem at the diagnosis, particularly in the context of breast cancer. However, breast fistulization or that of axillary lymphadenopathy are associated with a pseudoplastic nodular or scirrhous tumor of the breast, which progresses slowly, is strongly suggestive of breast tuberculosis. It is the histological and bacteriological study that confirms the diagnosis. Tuberculosis chemotherapy remains the mandatory therapeutic component. Surgical treatment might be necessary and has limited indications. The prognosis of mammary tuberculosis is generally good, but it provides that the treatment is well adapted with strong adherence to the treatment by the patient.

### What is known about this topic

- In Morocco, as an endemic country for tuberculosis, nearly 30,000 cases are recorded each year, which includes new cases and relapse cases. The incidence rate is around 87 cases per 100,000 habitants of which pulmonary tuberculosis represents half, while breast tuberculosis is a rare localization of extra-pulmonary tuberculosis, especially as a primary manifestation;
- The clinical presentation of breast tuberculosis is generally poorly described in the literature and clinically important features are not consistently reported;
- Anti-tuberculosis chemotherapy remains the mandatory therapeutic component.

### What this study adds

- Diagnosis remains difficult, as it can simulate a large number of much more common conditions, especially in older women where breast cancer remains the primary concern. The clinical and radiological pictures are often misleading and pose a real problem of diagnosis, particularly in the context of breast cancer. The article specifies the epidemiological, clinical, paraclinical, therapeutic and progressive characteristics of mammary tuberculosis and highlights the difficulties in diagnosis and management;
- The frequency of breast involvement is low and is seen mainly during the period of genital activity;
- As for the therapeutic aspect, the rate of drug-resistant tuberculosis is very low and surgery may be necessary and has limited indications.
